# Supplementary material for: Phylogenetic and Structure-Function Analyses of ENA ATPases: A Case Study of the ENA1 Protein from the Fungus Neurospora crassa
Source: Int J Mol Sci. 2023 Dec 30;25(1):514. doi: 10.3390/ijms25010514 (PMC10779151; doi:10.3390/ijms25010514)
Supplement: Supplementary file 1 [file ijms-25-00514-s001.zip › Fig. S1.pdf]

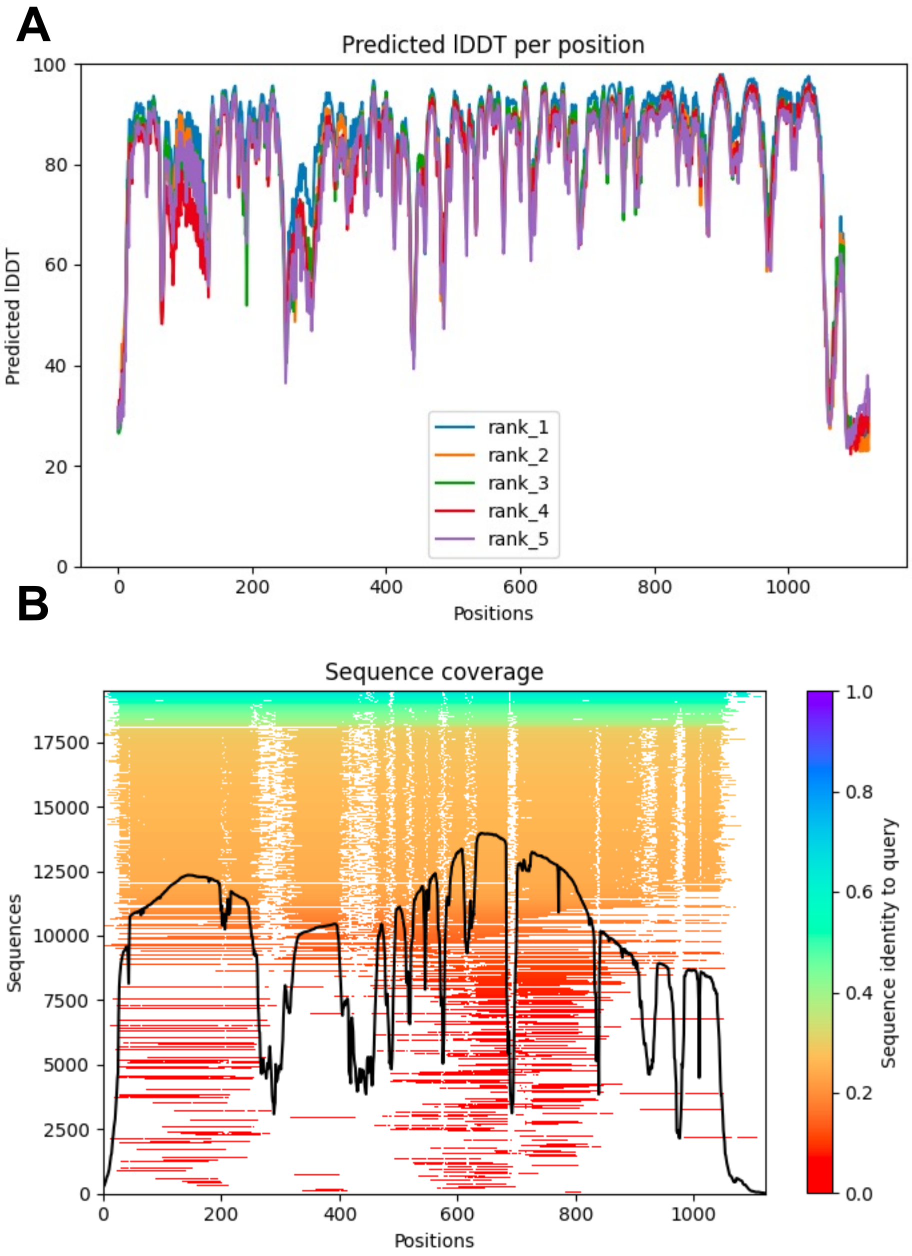

Fig. S1. NcENA1 (CAB65298.1) structure prediction metrics provided by AlphaFold2. A: pLDDT per residue obtained for each of the five models obtained, ordered from highest to lowest by the overall pLDDT value. **B:** MSA constructed using MMSeqs2 for prediction. Of the five structure models obtained, the one named as 'rank_1' was chosen for having the highest global pLDDT value.
