# Supplementary material for: Phylogenetic and Structure-Function Analyses of ENA ATPases: A Case Study of the ENA1 Protein from the Fungus Neurospora crassa
Source: Int J Mol Sci. 2023 Dec 30;25(1):514. doi: 10.3390/ijms25010514 (PMC10779151; doi:10.3390/ijms25010514)
Supplement: Supplementary file 1 [file ijms-25-00514-s001.zip › Fig. S2.pdf]

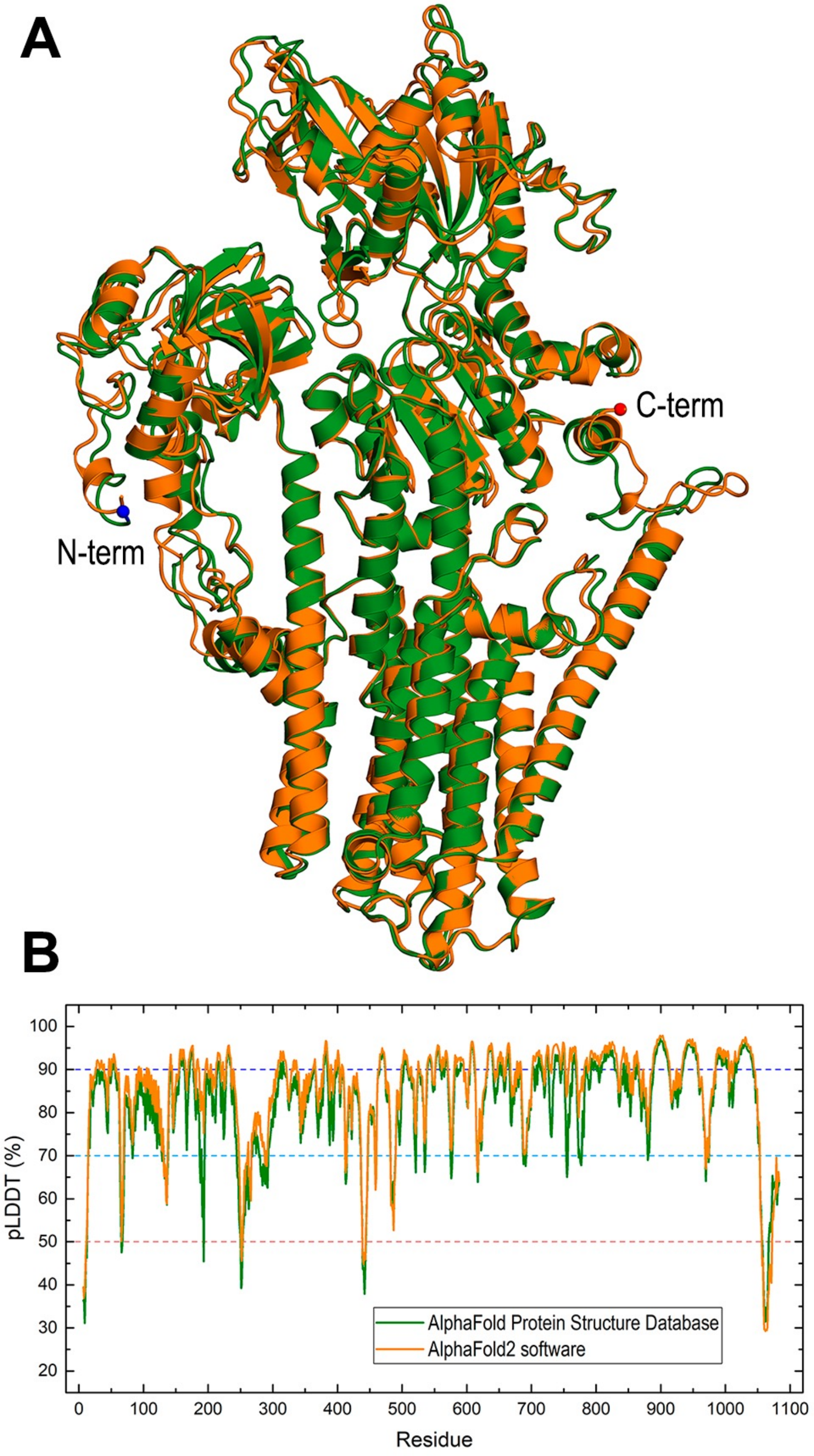


**Fig. S2. Comparison of two AlphaFold structural models of NcENA1 protein. A**: Superposition of both models. Green: AlphaFold Protein Structure Database, orange: obtained with AlphaFold2 software in this work. **B**: Plots of per-residue pLDDT metrics for both models. Horizontal dashed lines indicate thresholds for the confidence levels prescribed by AlphaFold as follows. Very high: > 90; high: between 90 and 70; low: between 70 and 50; very low: < 50.
