# Supplementary material for: Phylogenetic and Structure-Function Analyses of ENA ATPases: A Case Study of the ENA1 Protein from the Fungus Neurospora crassa
Source: Int J Mol Sci. 2023 Dec 30;25(1):514. doi: 10.3390/ijms25010514 (PMC10779151; doi:10.3390/ijms25010514)
Supplement: Supplementary file 1 [file ijms-25-00514-s001.zip › Fig. S3.pdf]

```
                                            TM4
                                             ** * *
NcENA1         328     EVI------------IYAVATGLSMIPASLVVVLTITMAAGT  370
SpCTA3         311     EVS------------IYAISLGISIIPESLIAVLSITMAMGQ  353
MpENA1         273     DII------------IYAVSLGIAVIPEGLIAVITIVMALGV  315
Na,K 3KDP_1    292     WLEA----------VIFLIGIIVANVPEGLLATVTVCLTLTA  334
H,K 7W4A_1     327     FLRA----------MVFFMAIVVAYVPEGLLATVTVCLSLTA  369
Na,K 2ZXE_1    322     WLEA----------VIFLIGIIVANVPEGLLATVTVCLTLTA  364
CaSERCA 1XP5_1 282     PVHGGSWIRGAIYYFKIAVALAVAAIPEGLPAVITTCLALGT  324
CaSERCA 7E7S_1 282     PVHGGSWIRGAIYYFKIAVALAVAAIPEGLPAVITTCLALGT  324
CaSERCA 2DQS_1 283     PVHGGSWIRGAIYYFKIAVALAVAAIPEGLPAVITTCLALGT  325
                        :                :.  :: :* .* ..::  ::

                                    TM5
                                      *  *
NcENA1         804     IFDNIQKFVLHVLAENIAQAGTLLIGLA  832
SpCTA3         754     MFDNIMRFVLHLLVSNVGEVILLVVGLA  782
MpENA1         711     IFSNIRKFILHLVSTNVGEVIVLIIGLA  739
Na,K 3KDP_1    743     IFDNLKKSIAYTLTSNIPEITPFLIFII  771
H,K 7W4A_1     778     IFDNLKKSIAYTLTKNIPELTPYLIYIT  806
Na,K 2ZXE_1    773     IFDNLKKSIAYTLTSNIPEITPFLVFII  801
CaSERCA 1XP5_1 753     IYNNMKQFIRYLISSNVGEVVCIFLTAA  781
CaSERCA 7E7S_1 752     IYNNMKQFIRYLISSNVGEVVCIFLTAA  780
CaSERCA 2DQS_1 754     IYNNMKQFIRYLISSNVGEVVCIFLTAA  782
                       ::.*: : : : :  *: :    .:

                               TM6
                           *  **
NcENA1         848     IVWIIMITSGLPDMGLGF  866
SpCTA3         798     ILWCNMITSSFPSMGLGM  816
MpENA1         755     ILFMNMVTSTPPAMALGV  773
Na,K 3KDP_1    782     ILCIDLGTDMVPAISLAY  800
H,K 7W4A_1     817     ILFIELCTDIFPSVSLAY  835
Na,K 2ZXE_1    812     ILCIDLGTDMVPAISLAY  830
CaSERCA 1XP5_1 792     LLWVNLVTDGLPATALGF  810
CaSERCA 7E7S_1 791     LLWVNLVTDGLPATALGF  809
CaSERCA 2DQS_1 793     LLWVNLVTDGLPATALGF  811
                       ::   : *.  *  .*.

                               TM7

NcENA1         890     IIDMIFYGLWITALCLASFV  910
SpCTA3         840     IVDMMVYGFFLGVVSLMTWV  860
MpENA1         795     LADMMVYGIIMGSLILVDWV  815
Na,K 3KDP_1    824     LISMAYGQIGMIQALGGFFT  844
H,K 7W4A_1     859     LAAYSYFQIGAIQSFAGFTD  879
Na,K 2ZXE_1    854     LISMAYGQIGMIQALGGFFS  874
CaSERCA 1XP5_1 834     FFRYMAIGGYVGAATVGAAA  854
CaSERCA 7E7S_1 833     FFRYLAIGCYVGAATVGAAA  853
CaSERCA 2DQS_1 835     FFRYMAIGGYVGAATVGAAA  855
                       :

                                       TM8
                                        *
NcENA1         948     WFALFLAWELVDMRRSFFRMQ--PGSKKYFTQ  980
SpCTA3         898     FCILIMAVEVKNFDNSLFNLHGIPWGEWNFRY  930
MpENA1         854     WMLLLHAYNCRHPRASLFTAEGGGASK------  986
H,K 7W4A_1     923     YQQYTCYTVFFISIEMCQIADVLIRKTRRLSA  955
Na,K 3KDP_1    888     IVEFTCHTPFFVTIVVVQWADLVICKTRRNSV  920
Na,K 2ZXE_1    918     IVEFTCHTSFFISIVVVQWADLIICKTRRNSI  950
CaSERCA 2DQS_1 892     FEAPEPMTMALSVLVTIEMCNALNSLSENQSL  924
CaSERCA 7E7S_1 890     FESPYPMTMALSVLVTIEMCNALNSLSENQSL  922
CaSERCA 1XP5_1 891     FEAPEPMTMALSVLVTIEMCNALNSLSENQSL  923
```

Fig. S3. Alignment of the TM4-8 helices of P2-type ATPases. The sequences of three ENA ATPases (NcENA1, SpCTA3 and MpENA1), three P2A-type (2DQS_1, 7E7S_1,1XP5_1) and three P2C-type (3KDP_1, 2ZXE_1,7W4A_1) ATPases were chosen for aligment. Residues previously identified as being involved in cation binding in other P2-type ATPases are marked in green with an asterisk. Yellow indicates residues that have been mutagenized in NcENA1 and have shown a change in function.
