# Supplementary material for: Phylogenetic and Structure-Function Analyses of ENA ATPases: A Case Study of the ENA1 Protein from the Fungus Neurospora crassa
Source: Int J Mol Sci. 2023 Dec 30;25(1):514. doi: 10.3390/ijms25010514 (PMC10779151; doi:10.3390/ijms25010514)
Supplement: Supplementary file 1 [file ijms-25-00514-s001.zip › manuscript.v9def.pdf]

International Journal of *Molecular Sciences*

MDPI

Article

Phylogenetic and Structure-Function Analyses of ENA ATPases: A Case Study of the ENA1 Protein from the Fungus *Neurospora crassa*

Marcos Aguilella [1,2], Blanca Garciadeblás [2], Luis Fernández Pacios [1,2,]* and Begoña Benito [1,2,]*

[1] Centro de Biotecnología y Genómica de Plantas (CBGP), Universidad Politécnica de Madrid (UPM), Instituto Nacional de Investigación y Tecnología Agraria y Alimentaria (INIA), Campus Montegancedo UPM, Pozuelo de Alarcón, 28223 Madrid, Spain; marcos.aguilella.3n@gmail.com

[2] Departamento de Biotecnología-Biología Vegetal, Escuela Técnica Superior de Ingeniería Agronómica, Alimentaria y de Biosistemas, Universidad Politécnica de Madrid (UPM), 28040 Madrid, Spain; b.garciadeblas@upm.es

* Correspondence: luis.fpacios@upm.es (L.F.P.); begona.benito@upm.es (B.B.)

Abstract: ENA transporters are a group of P-type ATPases that are characterized by actively moving Na^+ or K^+ out of the cell against their concentration gradient. The existence of these transporters was initially attributed to some fungi, although more recently they have also been identified in mosses, liverworts, and some protozoa. Given the current increase in the number of organisms whose genomes are completely sequenced, we set out to expand our knowledge about the existence of ENA in organisms belonging to other phylogenetic groups. For that, a hidden Markov model profile was constructed to identify homologous sequences to ENA proteins in protein databases. This analysis allowed us to identify the existence of ENA-type ATPases in the most primitive groups of fungi, as well as in other eukaryotic organisms not described so far. In addition, this study has allowed the identification of a possible new group of P-ATPases, initially proposed as ENA but which maintain phylogenetic distances with these proteins. Finally, this work has also addressed this study of the structure of ENA proteins, which remained unknown due to the lack of crystallographic data. For this purpose, a 3D structure prediction of the NcENA1 protein of the fungus *Neurospora crassa* was performed using AlphaFold2 software v2.3.1. From this structure, the electrostatic potential of the protein was analyzed. With all these data, the protein regions and the amino acids involved in the transport of Na^+ or K^+ ions across the membrane were proposed for the first time. Targeted mutagenesis of some of these residues has confirmed their relevant participation in the transport function of ENA proteins.

Keywords: ENA ATPases; Na^+ transporters; Alphafold2; *Neurospora crassa*; phylogeny

Citation: Aguilella, M.; Garciadeblás, B.; Fernández Pacios, L.; Benito, B. Phylogenetic and Structure-Function Analyses of ENA ATPases. A Case Study of the ENA1 Protein from the Fungus *Neurospora crassa*. *Int. J. Mol. Sci.* **2024**, *25*, x. https://doi.org/10.3390/xxxxx

Academic Editor: Stathis Frillingos

Received: 28 November 2023
Revised: 26 December 2023
Accepted: 27 December 2023
Published: date

Copyright: © 2023 by the authors. Submitted for possible open access publication under the terms and conditions of the Creative Commons Attribution (CC BY) license (https://creativecommons.org/licenses/by/4.0/).

1. Introduction

The ability to exchange molecules with the environment and respond to changes in environmental conditions is vital for the survival of any organism. This property is performed by a series of receptor and/or ion- or molecule-transporting proteins that are immersed in lipid bilayers. One of the protein families that provides the capacity to transport ions across cell membranes is the so-called P-type ATPases, a family of transporters that uses the energy of ATP hydrolysis to transport cations such as Na^+, K^+, Ca^{2+}, Cu^{2+}, or H^+, among others, and phospholipids against their concentration gradient [1]. The designation of P-type ATPases derives from the fact that during the catalytic cycle of protein activity, a transient intermediate is formed by phosphorylating an aspartate residue [2]. Traditionally, the transport cycle is described very simplistically according to the classical

Commented [M1]: ***IMPORTANT***
Please check your manuscript *carefully* and ***thoroughly.*** Changes are not allowed after proofreading. Language and the layout of this manuscript have been edited according to our publication standard.
Please do not delete comments.
Confirm (or revise) comments for the whole paper. Use a change tracking system or highlight when making adequate changes. Thank you. ... [1]

Commented [b2R1]: Authorship is OK in the ... [2]

Commented [EE3]: Attention: title altered, p ... [3]

Commented [b4R3]: Title corrected is fine. T ... [4]

Commented [M5]: Please carefully check the ... [5]

Commented [b6R5]: OK. the affiliations hav ... [6]

Commented [M7]: Please check if the provid ... [7]

Commented [b8R7]: I have checked the ... [8]

Commented [M9]: We changed dot/hyphen ... [9]

Commented [b10R9]: Confirmed

Commented [M11]: The provided informat ... [10]

Commented [b12R11]: I have changed the ... [11]

Commented [M13]: We moved "Pozuelo de ... [12]

Commented [b14R13]: OK.

Commented [M15]: We added the email ... [13]

Commented [b16R15]: OK

Commented [M17]: We added full name of ... [14]

Commented [b18R17]: OK

Commented [M19]: We rearranged ... [15]

Commented [b20R19]: OK. Dr. Luis Fernán ... [16]

Commented [M23]: 1. Please check through ... [19]

Commented [b24R23]: OK: gene symbols a ... [20]

Commented [M21]: Please check all author ... [17]

Commented [b22R21]: All author names ar ... [18]

Albers-Post model [3,4], in which these proteins are considered to undergo major conformational changes coupled to ion translocation. According to this model, P-ATPases can be found in two distinct conformational states: E1 and E2, each showing different affinity for the nucleotide and the transported ion.

P-type ATPases are relatively large, ubiquitous, and diverse membrane proteins involved in many transport processes in virtually all living organisms. They are characterized by sharing a highly conserved structural organization and a set of sequence motifs involved in their mechanism of action and function. The Ca^{2+}-ATPase SERCA1a (from Sarco (Endo)plasmic Reticulum CAlcium) from rabbit muscle was the first P-type ATPase in which its crystal structure was determined [5], and today we have extensive information on the structure of this pump in various conformations [6]. Later, structures of other P-type pumps also emerged, including the Na^+/K^+-ATPase [7] and the plasma membrane H^+-ATPase [8]. Despite a relatively low degree of sequence conservation, the structures of these pumps are strikingly similar, which allows the reliable prediction of model structures of P-ATPases on the basis of available experimental structural information. P-type ATPases are multidomain proteins that feature a transmembrane domain consisting of between 6 and 13 transmembrane (TM) helices and three cytoplasmic domains: the A domain (Actuator), the N domain (Nucleotide binding), and the P domain (Phosphorylation domain) [6,9]. The A domain is located between the TM2 and TM3 helices and forms a flexible globular domain that allows it some rotation. This domain contains one of the highly conserved sequence motifs of P-ATPases, the TGES motif, which stabilizes the transition between the two conformational states and participates in the hydrolytic dephosphorylation of aspartate mediated by a water molecule [9]. The P domain is located between the TM4 and TM5 helices and contains the conserved motif DKTG, whose first amino acid is the aspartate residue that is phosphorylated. Other conserved motifs in this domain are DPPR, TGDN, and GDGxND, which are involved in Mg^{2+} coordination, ATP binding, and aspartate phosphorylation. Finally, the N domain is inserted in the middle of the P domain and is responsible for ATP binding and phosphorylation of the P domain. This domain contains the conserved KGAPE motif [1].

Based on sequence homology, phylogenetic studies of P-type ATPases have identified five subfamilies (named P1-P5), characterized by substrate specificity and sequence motifs specific to each group: The P1 subfamily transports metals, such as Cu^{2+} or Cd^{2+}; the P2 subfamily includes Ca^{2+}, Na^+, Mg^{2+} or K^+ transporters; the P3 subfamily includes H^+ ATPases; the P4 subfamily transports phospholipids, being involved in the maintenance of lipid membrane asymmetry; and, finally, the P5 subfamily, whose proteins seem to be involved in the regulation of cation homeostasis in the endoplasmic reticulum [10,11], although their specificity is still unknown. Subfamilies P1, P2, P3, and P5 are further subdivided into eleven additional classes [10,12].

Within the subfamily of P2 ATPases are the so-called ENA-type proteins. These transporters belong to the P2D class, closely related to the endoplasmic reticulum (P2A), membrane (P2B) Ca^{2+} pumps, and the Na^+/K^+ pumps (P2C). ENA (from *Exitus NAtru*, Latin for "sodium exit") ATPases were first discovered in the yeast *Saccharomyces cerevisiae* [13], where their activity and substrate specificity were determined [14]. Unlike the Na^+/K^+ of the P2C group, which exchanges Na^+ (outward) for K^+ (inward), ENA ATPases are characterized by their capacity to transport Na^+ and/or K^+ to the cell exterior, thus relieving an excessive accumulation of these cations in the cytoplasm. Until now, the lack of experimental structural data on ENA proteins have prevented us from gaining a deeper understanding of the molecular mechanisms of their functions.

ENA proteins can actively extrude Na^+ (and/or K^+) against their concentration gradient, which confers to their host organisms the ability to survive in saline environments, even in alkaline pH conditions, where other Na^+ transport mechanisms such as Na^+-H^+ antiporters fail, being dependent on an H^+ concentration gradient. The existence of these transporters was initially attributed to some fungi, although more recently they have also been identified in mosses, liverworts, and some protozoa. However, ENA proteins are not

found in higher organisms such as plants or animals, which could potentially make them a good target for inhibitory drugs to control pathogenic protozoa and fungi. Systematic functional studies of different fungal ENA ATPases have shown that some ENA ATPases are specific for Na^+ efflux (such as NcENA1 from *Neurospora crassa* [15]), while others show poor discrimination between Na^+ and K+ or even that some might be specific for K^+ efflux (the best known example of the latter is CTA3 from *Schyzosaccharomyces pombe* [16,17]. Transcriptional studies confirm that these proteins are expressed in the presence of high salt concentrations and at an alkaline pH [17]. By obtaining yeast defective mutants in the genes encoding these proteins, it has been possible to demonstrate that ENA proteins are essential for organisms that live exposed to saline environments, maintaining a low cytoplasmic concentration of Na^+ to avoid its toxic effect and thus allowing their survival [18]. Saline media are both salinized soils widespread on the planet and marine environments, where a NaCl concentration of 500 mM is reached, as well as the plasma serum in which animal cells bathe (150 mM NaCl). This means that the activity of ENA proteins allows the proliferation and survival of organisms that possess them in extensive and very diverse environments.

ENA-type ATPases are widely distributed among fungi, although homologous proteins have also been identified in a group of primitive plants such as mosses (e.g., *Physcomitrella patens* [19]) and liverworts (*Marchantia polymorpha*), as well as in various protozoa such as *Trypanosoma* or *Leishmania*. However, ENA genes have not been found in higher organisms, such as vascular plants or animal cells [17]. This fact allows exploring the possibility of a dual biotechnological application of ENA proteins: as a target of action of drugs inhibiting their activity for the control of pathogenic protozoa and fungi [20], as well as using this type of transporter to increase tolerance to salinity in plant cultures through their heterologous expression [17]. On the other hand, the identification of ENA proteins in such diverse groups of organisms makes it interesting to conduct an in-depth phylogenetic study to find out the extent of these proteins in evolution. Pursuing these goals justifies extending the field of research on this group of transporters.

The aim of this work has been to perform a phylogenetic study of ENA proteins to update their evolutionary coverage. This study has been carried out by building a profile of a hidden Markov model and Bayesian inference. In addition, the prediction of the three-dimensional structure of the NcENA1 protein of the fungus *N. crassa* using AlphaFold2 has been addressed. Finally, a more exhaustive study of the structure and function of this protein has been conducted, trying to propose possible relevant amino acids involved in the formation of the specific channel through which Na^+ and/or K^+ are transported. By site-directed mutagenesis of some of the proposed amino acids and expression of the mutated ENA protein in yeast, we have been able to verify their role in protein function.

2. Results

2.1. Identification of ENA Proteins in New Groups of Organisms Using the Hidden Markov Model

To precisely determine the groups of organisms that possess ENA-type P-ATPases, an exhaustive search of these proteins in the currently available databases were proposed. For this purpose, a profile of a hidden Markov model defining the conserved regions of ENA proteins was constructed (see Materials and Methods). HMM profiles are a description of the consensus sequence of an MSA. These profiles use a position-specific scoring system to capture information about the degree of conservation at various positions in the multiple alignments. This fact makes it a much more sensitive and specific method for searching homologous sequences in databases than pairwise methods, such as those used by BLAST, which use position-independent scoring [23]. Once the HMM profile for ENA proteins was proven, it was used to search for novel ENA sequences by pitting the HMM profile against protein sequence databases such as the Joint Genome Institute (JGI)

Commented [M25]: Please note:
For IJMS, Article paper should follow the structure of:
1. Introduction;
2. Results;
3. Discussion (2 and 3 can be combined 2. Results and Discussion);
4. Methods and Materials;
5. Conclusions (optional)
We have changed the order of the sections, please check and make sure the reference, figure, table citation are still in numerical order.
If it is not convenient for you to reorder the references, please ensure that all the references are correctly cited in text, we can help rearrange the references after the proof.

Commented [b26R25]: When changing the order of sections it is necessary to change the reference numbers and I find this difficult to do at this stage of the revision. The references are well cited in the text but I would appreciate your help with the renumbering task.

(https://genome.jgi.doe.gov/portal/pages/tree-of-life.jsf, v8.18.170, last accessed 18 October 2023), NCBI (https://www.ncbi.nlm.nih.gov/, last accessed 18 October 2023) [47], and Uniprot (https://www.uniprot.org/, last accessed 18 October 2023) [48].

The analysis corroborated what was already known: that ENA proteins do not exist in prokaryotic organisms and that the existence of these proteins is limited to some phylogenetic groups of eukaryotes. Among eukaryotes, new ENA proteins were identified within the fungal group (Figure 1 and Table S1 of Supplementary Material).

Although the existence of ENA proteins in this group was already known, the present study has allowed the identification of new homologous proteins that exist in phylogenetically more primitive groups of fungi, such as some belonging to the phylum Chytridiomycota and Zoopagomycota. Nonetheless, there are organisms currently classified within the group of fungi, such as those included in Cryptomycota and Microsporidia, as well as a group of the phylum Chytridiomycota classified as Chytridiomycetes, that do not seem to possess ENA ATPases. On the other hand, the search for ENA proteins within the Viridiplantae group confirmed the existence of ENA proteins in the Chlorophyta group and in primitive plants such as mosses and liverworts other than those previously identified, although they do not exist in Anthoceros, a group phylogenetically very close to both. This search has also confirmed that ENA proteins do not exist in higher plants. The non-identification of ENA ATPases in some organisms could be due to an incomplete or inappropriate annotation of the proteomes of these organisms, but it could rather indicate that throughout evolution and probably depending on the environmental conditions in which each organism has thrived, it has been selected to either have or not have certain mechanisms that fulfill specific physiological functions. Interestingly, the screening of ENA ATPases in other phylogenetic groups of eukaryotes showed their existence in new organisms belonging to the groups Heterobolosea, Heterokonta (also known as stramenopiles), and Rhizaria (Figure 1 and Table S1).

From the results obtained in the search for proteins homologous to ENA ATPases, it was noteworthy that, although ENA representatives appeared within the phylum Euglenozoa, which includes the protozoa of genera *Trypanosoma*, *Leishmania*, and *Naegleria* that were already known to possess these proteins (Figure 1 and [17]), no homologues appeared in the pathogenic protozoan *Plasmodium*. This result was striking because it has recently been published that the *Plasmodium falciparum* parasite possesses a Na^+ transporting ENA-type ATPase called PfATP4, which, in fact, is currently being intensively investigated as a possible target of action against this pathogen [49]. To clarify this discrepancy, *Plasmodium* proteins were subjected to ENA protein HMM profiling along with other proteins identified in other organisms that showed homology to that of *Plasmodium*, which had also recently been proposed as ENA-type ATPases [50] (included in Table S1). Surprisingly, the HMM profile returned a non-zero ‘e-value’, greater than 8.7×10^{-212}, which seemed to suggest that none of these proteins were ENA ATPases.

Commented [M27]: We revised scientific notation format, please confirm.

Commented [b28R27]: Confirmed. Thank you.

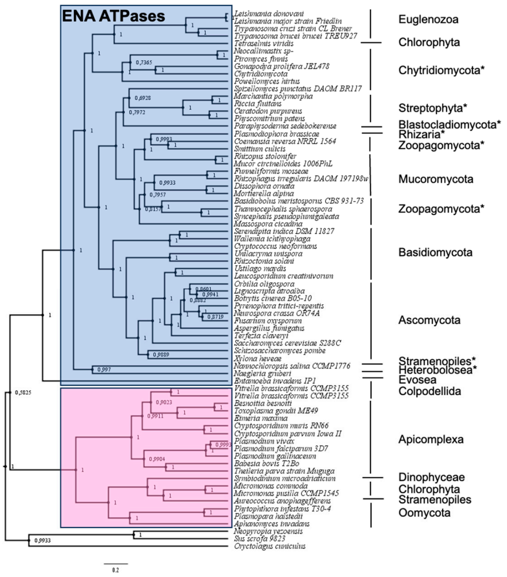

Figure 1. Phylogenetic tree of ENA ATPases. It was based on the alignment of 72 sequences compiled in Table S1 (Supplementary Material), which include known and newly identified ENA ATPases using the Hidden Markov Model (labeled with blue background), along with 'proposed as' ENA proteins in [50] (labeled with pink background). Three P2-type P-ATPases other than ENA proteins are included as an outgroup. Phylogenetic groups in which ENA ATPases have been identified for the first time are marked with asterisks. Numbers at nodes indicate the probability of Bayesian inference. The phylum to which the different organisms belong is indicated on the right.

2.2. Phylogenetic Analysis of 'Newly Identified' and 'Proposed as' ENA ATPases

To find out whether the proposed P-ATPases of *Plasmodium* as well as those published in [50] were truly ENA ATPases, a phylogenetic comparison with other subfamilies of P-type ATPases (P1 to P5) was performed (Figure 2 and Table S2, which contain information on the proteins chosen for comparison).

Commented [M29]: We moved Figure 1 here to keep references in numerical order, please confirm.

Commented [b30R29]: The reference numbers will have to be modified due to the change of position of the "Results" sections to "Materials and Methods" (I rely on your help for this), so it is not clear if the new position of Fig. 1 will be better.

Commented [M31]: Please use periods as decimal signs instead of commas, e.g., "0,1" should be "0.1".

Commented [b32R31]: In Figure 1, the data have been kept as generated by the program, with commas instead of dots.

Commented [M33]: Please confirm if the bold should be retained.

Commented [b34R33]: Yes, it is. The bold should be retained.

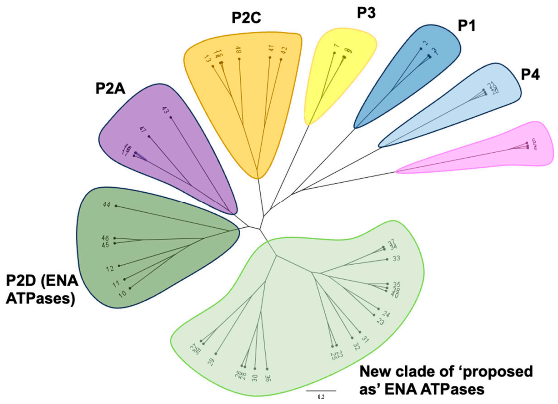

Figure 2. A phylogenetic tree showing the main clades of P-type ATPases. Its construction was based on the alignment of 48 proteins representative of the main clades compiled in Table S2 (Supplementary Material). The main classes of ATPases are distinguished by different colors: ENA ATPases (P2D, in dark green), SERCA ATPases (P2A, purple), Na/K ATPases (P2C, orange), H^+ ATPases (P3, yellow), heavy metal ATPases (P1, dark blue), ER ATPases (P5, pink), and phospholipid ATPases (P4, light blue) groups are highlighted. The new clade of 'proposed as' ENA ATPases is marked in light green.

Special attention was paid to the choice of proteins representing the P2 subfamily in addition to the ENA ATPases (2D), such as the Ca^{2+}-SERCA ATPases (2A) and the Na^+/K^+ ATPases (2C), with which the questioned ATPases could have more sequence similarity. In the selection of ATPases from the different subfamilies to compare, it was also considered to choose representatives belonging to protist organisms to reduce the bias derived from the organism from which the proteins originate. The inferred phylogenetic tree showed the existence of a new clade including the proteins initially proposed as ENA ATPases that is perfectly differentiated from the ENA and from the rest of the P-type ATPases clades (Figure 2). Interestingly, the location within the clade of ENA proteins of proteins from protist organisms that belong to the Euglenozoa, Stramenopiles, Heterobolosea, and Evosea groups allows us to rule out that the separation into two clades is due to the evolutionary separations that exist between these eukaryotic organisms and fungi.

If we compare the phylogenetic distances between the proteins involved in Na+ transport, i.e., ENA ATPases, Na^+/K^+ ATPases, and those forming the new clade [49,50], we observe that the latter are closer to ENA-type ATPases than to Na^+/K^+ ATPases. Therefore, it should be noted that, within the group of protists, there are organisms that possess typical ENA ATPases that are located in the ENA ATPase clade (sequences in Figure 2 numbered from 44 to 46) and others that possess a new type of P-ATPases that would form a separate clade (Figure 2, from 22 to 40), which would allow extending the diversity of P-ATPases with Na^+ transport capacity.

2.3. *Prediction of NcENA1 ATPase Structure*

To have a model of the 3D structure of ENA ATPases, the prediction was made with AlphaFold2 software v2.3.1, using as an example the sequence of the ENA1 protein from the fungus *Neurospora crassa* (NcENA1). The complete sequence of this protein has 1121 amino acids and is known to be an efficient Na+ efflux system, although it also transports K^+ to the outside with lower efficiency [15,17].

Commented [M35]: Digits in figure are overlapped, please revise and ensure that all contents in the figure are clearly visible and do not affect scientific reading.

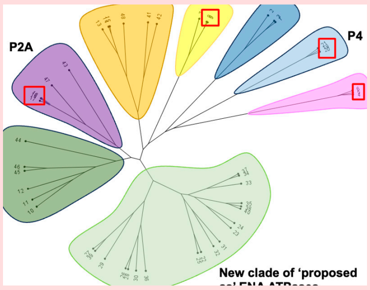

Commented [b36R35]: The figure we have is an image and we cannot modify the position of the digits to avoid overlapping. We consider that with the current aspect, the numbers can be visualized well and could look like this. Thank you.

Commented [M37]: Please confirm if the bold should be retained.

Commented [b38R37]: Yes, it is. The bold should be retained.

Commented [M39]: Please state the version number of the software. If not available, please be sure to provide the URL link: URL (accessed on day month year).

Commented [b40R39]: The version of AlphaFold software used is v2.3.1. Now is included in the text

Among the five structure models provided by Alphafold2 for the NcENA1 protein, the one with the highest overall pLDDT value, 83.3%, was chosen, revealing high confidence in the model [28,34] (Figure S1). The comparison of this model with that included in the AFPSDb is shown in Figure S2. According to that recommended by the AlphaFold authors for end protein segments displaying especially low pLDDT values [28], the two short disordered segments N-terminal 1-6 and C-terminal 1084–1121 (that have per-residue pLDDT < 30%) were discarded in both models. Not only does the structural alignment demonstrate their close agreement (RMSD = 1.1 Å over the 1077 residues, Figure S2A), but also their per-residue pLDDTs along the sequence reveal that they refer to the same structure (Figure S2B). It is worth mentioning that our model obtained with AlphaFold2 shows in many residues pLDDT values even slightly better than those of the corresponding residues in the AFPSDd model (Figure S2B). In fact, the number of residues with pLDDT values > 90% (confidence "very high") is 466 in our model and 288 in the AFPSDb model. The reason for this difference is that when the AFPSDb was enlarged to cover more than 200 million proteins, the options to generate the predicted structures were not as fine as those available to a user of the AlphaFold2 software v2.3.1 (in particular, the options associated with templates in the PDB and with the construction of the MSA) that are applied to predict the structure of a single protein. As a further test of agreement between both models, we computed their moments of inertia that resulted in essentially indistinguishable values (amu Å2): 20.308, 33.028, and 35.519 for our model, and 20.202, 33.233, and 35.532 for the AFPSDb model.

In what follows, all results refer to the model of the NcENA1 protein here obtained with Alphafold2. This model, shown in Figure 3, demonstrates structural conservation with P-type ATPases, clearly differentiating the three cytosolic domains typical of this family of proteins corresponding to the A, N, and P domains and the transmembrane domain composed of 10 transmembrane helices (Figure 3).

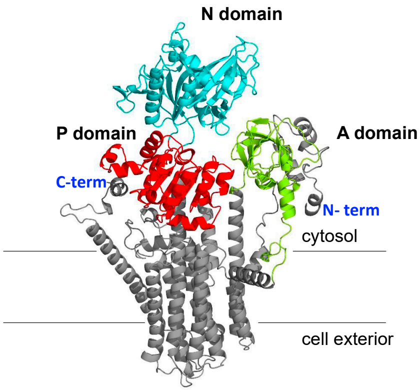

Figure 3. Three-dimensional structure model for the NcENA1 protein obtained with the AlphaFold2 software v2.3.1. The model is colored according to the cytosolic domains: A domain (lime green), P domain (red), and N domain (cyan). The rest of the protein is colored gray, corresponding to the TM helices and N- and C-termini of the protein. The lines indicate the approximate limits of the lipid membrane.

As mentioned in Materials and Methods, the number of residues with pLDDT values indicating "very high" (AlphaFold term) confidence (466 out of a total of 1077) is particularly high (Figure S1A). The MSA showed that many sequences (>17,500) were used for the predictions, most of which have a low identity of approximately 30%, corresponding

Commented [M41]: Please state the version number of the software. If not available, please be sure to provide the URL link: URL (accessed on day month year).

Commented [b42R41]: Done. I included "v2.3.1"

Commented [M43]: Please confirm if the bold should be retained.

Commented [b44R43]: Yes, it is. The bold should be retained.

to sequences of the P2-type ATPase subfamily for which crystallographic data are available (Figure S1B). This observation reinforces the reliability of the method. Despite the high number of sequences contained in the MSA, there were sequence segments that had low coverage and that coincided with decreases in per-residue pLDDT values (Figure S1A). These segments corresponded with those of the amino acid sequences most specific to P2D-type ENA ATPases that distinguish them from the other ATPases of the P2 subfamily, so the confidence of the prediction was diminished by not having enough reference crystallographic structures. Despite these local minor issues, the overall Alphafold2 metrics indicated that the modeled structure for the NcENA1 protein would be reliable, allowing further analyses of molecular properties such as electrostatic potentials as well as the prediction of possible Na^+ or K^+ ion binding sites.

2.4. Electrostatic Potentials and Possible Na^+ and/or K^+ Binding Sites of NcENA1

Electrostatic potential (EP) maps describe the spatial charge distributions of proteins, providing valuable information for assessing their interactions with other molecules, with ligands or ions, and with the aqueous environment. Electrostatics is the major force intervening in molecular interactions due to its long-range effect since attractions or repulsions arising from atomic charges decay with the inverse of the interaction distance. Furthermore, in the specific case of ion-moving transmembrane proteins, EPs also allow us to evaluate the influence of charged residues involved in the conductance of pathways followed by ions to cross membranes [51]. The PB-EP mapped onto the molecular surface of the NcENA1 protein is shown in Figure 4A.

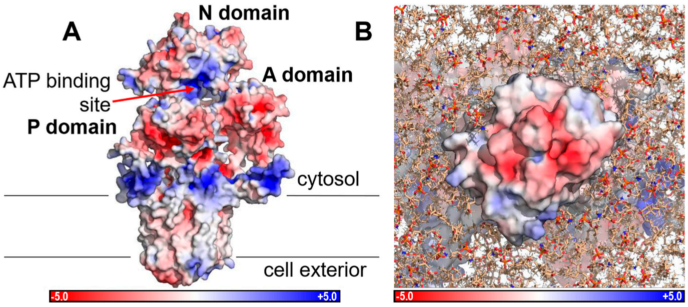

Figure 4. Poisson Boltzmann electrostatic potential (PB-EP) mapped onto the molecular surface of the complete NcENA1 protein ((**A**), side view) and of the extracellular part ((**B**), top view). Red colors indicate a negative PB-EP, while blue colors indicate a positive PB-EP according to the color bar below, whose units are kT/e (1 kT/(eÅ) = 2.59 × 108 V/m). The NcENA1 molecule is shown in the same orientation as in Figure 3. Lines in (**A**) indicate the approximate limits of the lipid membrane. The lipid membrane in (**B**) is constructed with the tools available in the CHARMM-GUI web (see Material and Methods in Section 4).

Note the positive electrostatic potentials of both ATP binding sites and membrane binding sites (Figure 4A). Electrostatic interactions help bind ATP in its binding site and anchor the protein correctly to the membrane by interacting, respectively, with the negative phosphates of ATP and with the negatively charged polar heads of the plasma membrane lipids. On the other hand, Figure 4B shows the PB-EP mapped onto the surface as seen from the cell exterior, in which a large surface with negative PB-EP stands out. This negative potential extends across the entire extracellular surface (Figure 4A, B) and coincides with the ends of the TM helices, thus acquiring great importance in the conductance of the ion channel through an electrostatic mechanism.

Commented [M45]: Please change the hyphen (-) into a minus sign (−, "U+2212"), e.g., "-1" should be "−1".

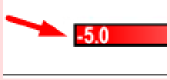

Commented [b46R45]: The software used to prepare this Figure does not permit using extra characters. The well-known property to which the scale refers has a negative and a positive range of values, so there is no possibility of ambiguity in using the hyphen sign, particularly when in the other end of the scale appears the "+" sign.

Commented [M47]: We added section citation, please confirm.

Commented [b48R47]: OK!! Confirmed

However, the ion exit site does not extend across this entire surface; rather, and according to previous studies on other P2 type P-ATPases [52], the ion channel seemed to be formed in the space between helices TM4, TM5, TM6, and TM8. To verify that ENA proteins retain a similar ion channel, we calculated the electric field lines, which indicate the path followed by a free electric charge moving in an electric field, as would be the case for Na^+ or K^+ ions. Examining the electric field lines of the extracellular part, shown in Figure 5, it can be observed how there are different zones with a high density of field lines, which indicates a greater magnitude of the electric field in these zones.

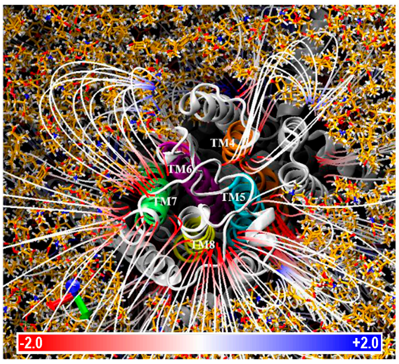

Figure 5. Electric field lines of the extracellular interface of the NcENA1 protein. Lines are shown on a scale of −2.0 to 2.0 kT/Å according to the color bar. TM4 (orange), TM5 (blue), TM6 (violet), TM7 (lime green), and TM8 (yellow) helices are highlighted. Note the high concentration of field lines in the space between the highlighted TM helices. Lipids (sticks with yellow carbons) of the membrane set using CHARMM-GUI as explained in Material and Methods are shown surrounding NcENA1 (gray cartoon).

One of the areas with high line density is the space formed between helices TM4, TM5, TM6, and TM8, indicating that ENA-type ATPases could conserve the same ion channel as other P-ATPases. However, there are other areas with high line density that, without further analysis, cannot be ruled out initially and might also be involved in the transport of ions across the membrane. To test whether these zones could be part of the ion pathway, two-dimensional slices of the PB-EP map were analyzed across the transmembrane helices along the axis perpendicular to the membrane. If they were part of the ion pathway, an electrostatic potential should be visualized along the entire transmembrane, through which Na^+ or K^+ ions could be guided. In the slices shown in Figure 6, it can be observed that at the cytoplasmic border of the membrane there are several points with a negative PB-EP, in particular those between helices TM6, TM7, and TM8, and those between TM4, TM5, and TM6 (Figure 6C), points that could indicate the entry of two Na^+ and/or K^+ atoms.

Commented [M49]: Please change the hyphen (-) into a minus sign (−, "U+2212"), e.g., "-1" should be "−1".

Commented [b50R49]: The software used to prepare this Figure does not permit using extra characters. The well-known property to which the scale refers has a negative and a positive range of values, so there is no possibility of ambiguity in using the hyphen sign, particularly when in the other end of the scale appears the "+" sign

Commented [M51]: Please confirm if the bold should be retained.

Commented [b52R51]: Yes, it is. The bold should be retained.

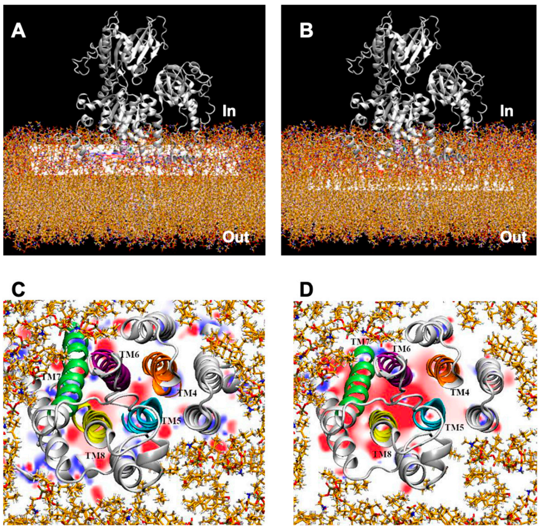

Figure 6. **Two-dimensional slices of the PB-EP cutting transmembrane helices.** TM4 (orange), TM5 (blue), TM6 (violet), TM7 (green), and TM8 (yellow) along the axis perpendicular to the membrane at the cytoplasmic border (**A**,**C**) and at the middle of the membrane (**B**,**D**). Red and blue colors in the slices indicate negative and positive PB-EPs, respectively, on a scale ranging from −20 to +20 kT/e.

Commented [M53]: Please confirm if the bold should be retained.

Commented [b54R53]: Yes, it is. The bold should be retained.

As one dives into the membrane, both the extent of the negative areas and the intensity of the negative PB-EP increase, thus creating a wide zone of negative potential. This would be instrumental for the conductance of positively charged ions (Na^+ or K^+) through the ion channel guided by a purely electrostatic mechanism. Therefore, after analyzing the electric field lines on the cell exterior and the electrostatic potential map slices, it appears that, despite having several zones with a high density of electric field lines, the ion channel of the ENA proteins can be formed by the TM4, TM5, TM6, TM7, and TM8 helices.

The different crystallized structures of endoplasmic reticulum calcium pumps (P2A-type ATPases) have allowed the identification of two Ca^{2+} binding sites: site I is composed of TM5, TM6, and TM8 helices residues, and site II is predominantly composed of TM4 residues [52]. The results just presented concerning the finding of an intense negative PB-EP between helices could indicate that the ENA proteins also conserve these binding sites. However, in addition to those four TM4, TM5, TM6, and TM8 helices, it is possible that some residues of the TM7 helix could also be part of the binding sites.

To explore possible Na^+ or K^+ binding sites in the predicted structure for NcENA1, two Na^+ cations were added in the center of the space left between TM4, TM5, TM6, TM7, and TM8 helices, followed by a potential energy minimization of the full protein-ions complex (see Material and Methods). The result of this minimization arranged the two sodium ions 5.6 Å apart from each other and placed them at precise locations that define two binding sites (Figure 7).

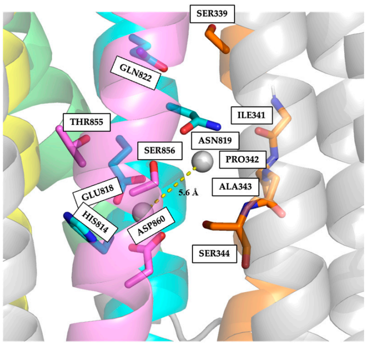

Figure 7. Possible Na⁺ binding sites of NcENA1. The TM4 (orange), TM5 (blue), TM6 (violet), TM7 (green), and TM8 (yellow) helices are highlighted, as are the side chains of the following residues: H814, E818, N819, and Q822 in TM5; T855, S856, and D860 in TM6; and S339, I341, P342, A343, and S344 in TM4. Na^+ ions are represented by the silver balls, separated by a distance of 5.6 Å, shown as a yellow dashed line.

Commented [M55]: Please confirm if the bold should be retained.

Commented [b56R55]: Yes, it is. The bold should be retained.

Site I would be formed by residues H814 and E818, belonging to the TM5 helix, and T855, S856, and D860 of the TM6 helix, all residues having electronegative oxygens that would provide the negative electrostatic potential. In particular, the two negatively charged acidic residues E818 and D860 would adopt a clamp conformation, maximizing their electrostatic attraction to the cations. Site II would be formed by the main chain oxygen atoms of I341, P342, A343, and S344, residues that form the conserved sequence motif "IPAS" of the TM4 helix, and the side chains of N819 and Q822 of TM5, both residues providing side chain electronegative oxygens.

A closer examination of the amino acid sequences of transported cation binding regions described in other P2-type ATPases for which crystallographic data are available compared with that of NcENA1 revealed clear differences in the residues involved (Figure S3). These differences were interesting and encouraged us to study the residues that might be implicated in the Na^+ and/or K^+ binding sites, as well as the specific channel through which they were transported.

2.5. Identification of Relevant Functional Residues in NcENA1 ATPase by Site-Directed Mutagenesis

To find out whether the proposed Na+ binding sites could be involved in the binding and transport of the cation across the membrane, some mutants were obtained (Table 1), and the effect of replacement of these amino acid residues on the function of Na+ transport was examined by heterologous expression of NcENA1 mutant proteins in the B3.1 strain of *S. cerevisiae*.

Table 1. Changes made in some NcENA1 residues by targeted mutagenesis and their effect on Na^+ and K^+ growth when expressed in B3.1 yeast mutants. Wild-type sequences (NcENA1(wt)) are shown in the blue background rows, indicating the transmembrane fragment where they are located. The modified sequences are shown in the white background rows, with the mutated residue in red. Growth in the presence of NaCl and KCl as well as the deduced ion efflux phenotype are described in the last three columns, respectively. Symbols ++, +, +, -, -- are used to indicate the level of growth observed in the culture media ranging from increased growth (++) to total absence of growth (--). ↑ or ↓ indicate increased or decreased growth relative to the wild type respectively.

Strain Name	Protein Sequence	TM Location	Growth in Na^+	Growth in K^+	Comments on Phenotype
**NcENA1(wt)**	**I P A S L**	TM4	++	+	**Na^+-efflux/slight K^+ efflux activity**
**A343E**	**I P E S L**	"	+	--	↓ Na^+-efflux/Loss of K^+ efflux
**S344G**	**I P A G L**	"	++	-	↓ K^+ efflux
**NcENA1(wt)**	**E N I A Q**	TM5	++	+	**Na^+-efflux/slight K^+ efflux activity**
**E818V**	**V N I A Q**	"	--	--	Loss of Na^+ and K^+ efflux function
**N819V**	**E V I A Q**	"	--	--	Loss of Na^+ and K^+ efflux function
**Q822V**	**E N I A V**	"	--	++	Loss of Na^+ efflux/ ↑ K^+ efflux
**NcENA1(wt)**	**T S G L P D**	TM6	++	+	**Na^+-efflux/slight K^+ efflux activity**
**T855V**	**V S G L P D**	"	--	--	Loss of Na^+ and K^+ efflux function
**S856A**	**T A G L P D**	"	+	--	↓ Na^+-efflux/Loss of K^+ efflux
**D860V**	**T S G L P V**	"	+	++	↓ Na^+-efflux/ ↑ K^+ efflux
**D860S**	**T S G L P S**	"	--	--	Loss of Na^+ and K^+ efflux function
**NcENA1(wt)**	**F I I D M I F Y**	TM7	++	+	**Na^+-efflux/slight K^+ efflux activity**
**D892V**	**F I I V M I F Y**	"	--	--	Loss of Na^+ and K^+ efflux function
**NcENA1(wt)**	**F L A W E L V D M R R S**	TM8	++	+	**Na^+-efflux/slight K^+ efflux activity**
**S963A**	**F L A W E L V D M R R A**	"	+	++	↓ Na^+-efflux/ ↑ K^+ efflux
**NcENA1(wt)**	**W E W G I V**	C-term	++	+	**Na^+-efflux/slight K^+ efflux activity**
**E1021V**	**W V W G I V**	"	--	--	Loss of Na^+ and K^+ efflux function

This yeast mutant is very sensitive to Na^+ and does not grow in the presence of salt in the medium because it lacks its ENA ATPases and the plasma membrane Na^+-H^+ antiporter NHA1 (see Materials and Methods). The Na^+-extrusion activity of the obtained NcENA1 mutants can be inferred from the level of salt-sensitivity complementation of the transformed B3.1 cells expressing the mutant genes. As shown in Figure 8 and Table 1, transformants expressing mutants A343E of TM4, S856A and D860V of TM6, and S963A of TM8 showed reduced salt tolerance compared to the wild-type NcENA1 gene.

Commented [M57]: please confirm if the bold should be retained.

Commented [b58R57]: Yes, we think that this way the items in the table are clearer, but if you think it is better to change it, please change it!

Commented [M59]: please add explanation for +, ++, -, --, ", and arrows.

Commented [b60R59]: You are right. Now the following explanation has been included: "The symbols ++, +, +, -, -- are used to indicate the level of growth observed in the culture media ranging from increased growth (++) to total absence of growth (--). Upward or downward arrows indicate respectively increased or decreased growth relative to the wild type"
