# Supplementary material for: Phylogenetic and Structure-Function Analyses of ENA ATPases: A Case Study of the ENA1 Protein from the Fungus Neurospora crassa
Source: Int J Mol Sci. 2023 Dec 30;25(1):514. doi: 10.3390/ijms25010514 (PMC10779151; doi:10.3390/ijms25010514)
Supplement: Supplementary file 1 [file ijms-25-00514-s001.zip › Table S3.pdf]

Table S3. Primers used in the present study to obtain NcENA1 mutants by targeted mutagenesis.

Primer name	Sequence 5´-3´
NcA343E-1	GTCGATGATTCCGGAGAGTTTGGTG
NcA343E-1R	CACCAAACTCTCCGGAATCATCGAC
NcS344G-1	TTCCGGCGGGTTTGGTGGTGGTGTT
NcS344G-1R	ACCAAACCCGCCGGAATCATCGACA
NcE818V-1	CGGTGAACATCGCTCAGGCAGGC
NcE818V-1R	CGATGTTCACCGCCAAGACGTGC
NcN819V-1	CGGAGGTCATCGCTCAGGCAGGC
NcN819V-1R	CGATGACCTCCGCCAAGACGTGC
NcQ822V-1	GAGAACATCGCTGTGGCAGGCAC
NcQ822V-1R	GGGTGCCTGCCACAGCGATGTTC
NcT855V-1	CATCATGATCGTCTCCGGTCTAC
NcT855V-1R	ACCGGAGACGATCATGATGATCC
NcD860S-1	CTCCGGTCTACCCTCCATGGGCC
NcD860S-1R	GGAGGGTAGACCGGAGGTGATCA
NcD892V-1	TCATCGTCATGATCTTTTACGGA
NcD892V-1R	GACGATGATGAATTCCAAAGTAA
NcS963A-1	CGCGCCTTCTTCCGGATGCAGCCTG
NcS963A-1R	GGCGCGGCGCATATCGACGAGCTCC
